# Structural Insight into the Tetramerization of an Iterative Ketoreductase SiaM through Aromatic Residues in the Interfaces

**DOI:** 10.1371/journal.pone.0097996

**Published:** 2014-06-05

**Authors:** Hua Wang, Huaidong Zhang, Yi Zou, Yanling Mi, Shuangjun Lin, Zhixiong Xie, Yunjun Yan, Houjin Zhang

**Affiliations:** 1 Key Laboratory of Molecular Biophysics, Ministry of Education, Wuhan, Hubei, China; 2 Department of Biotechnology, College of Life Science and Technology, Huazhong University of Science and Technology, Wuhan, Hubei, China; 3 State Key Laboratory of Microbial Metabolism, School of Life Sciences & Biotechnology, Shanghai Jiao Tong University, Shanghai, China; 4 College of Life Sciences, Wuhan University, Wuhan, Hubei, China; University of Oulu, Finland

## Abstract

In the biosynthesis of polyketides, ketoreductases (KRs) are an important group of enzymes that determine the chiralities of the carbon backbones. SiaM is a special member of this group that can recognize substrates with different lengths and can be used iteratively. Here we report the crystal structure of SiaM. Structural analysis indicates that the overall structure resembles those of other KRs. However, significant disparity can be found in the conserved LDD motif that is replaced with IRD motif in SiaM. The isoleucine and aspartic acid residues take similar orientations as leucine and aspartic acid in the conserved LDD motif, while the arginine residue points out towards the solvent. PISA analysis shows that SiaM forms a tetramer. Several aromatic residues are found in the interfaces, which have aromatic stacking interactions with the aromatic residues in the neighboring protomers. Mutagenesis studies performed on the aromatic residues show that these sites are important for maintaining the structural integrity of SiaM. However, the aromatic residues contribute differently to the enzymatic activity. In the N-terminal interface, the aromatic residues can be replaced with leucine without affecting the enzymatic activity while, in the other interface, such mutations abolish the enzymatic activity.

## Introduction

Many clinically important natural products are synthesized by large modular polyketide synthases (PKSs) [1], [2]. Each module in PKSs is responsible for one round of chain extension and subsequent modification. The nascent polyketide is then passed to next module to continue the elongation process. A functional module in PKSs harbors at least three domains: a ketosynthase (KS) domain, an acyltransferase (AT) domain and an acyl carrier protein (ACP) domain. They are responsible for the synthesis of the polyketide backbone [3]. In addition to these essential domains, most PKS modules contain one or more enzymes responsible for post-modification of β-keto carbons. The β-keto groups are reduced to β-hydroxyl groups with ketoreductases (KRs) [4]. Then dehydratases (DHs) dehydrate the β carbon to install a double bond that is reduced to a single bond with enoylreductases (ERs) [5].

Among these enzymes, the KRs are responsible for the chiralities at β-carbons. Recently, the studies of individual KR domains separated from their hosting PKSs have revealed a great amount of information on the stereochemistry of ketoreduction reactions [6], [7]. As the 4-pro-*S* hydride of cofactor NADPH is commonly used in ketoreduction reactions, it was proposed that the orientations of the polyketide substrates in the active site are the determining factors for the β-hydroxyl stereochemistry [8]. Also, some correlation can be found between conserved residues and stereochemistry of the products. Conserved LDD motifs are discovered in type-B KRs that produce β-hydroxyl groups with *R* stereochemistry, while a tryptophan is conserved in type-A KRs that produce β-hydroxyl groups with *S* stereochemistry [9]. The crystal structures of several KR domains show that the conserved catalytic triad KSY is positioned as those found in the other short-chain dehydrogenase/reductase (SDR) enzymes. The substrates are located close to the nicotinamide rings, which enables the 4-pro-*S* hydride to attack the β-keto group [10], [11]. The comparison of A-type and B-type KR domain structures has shown that the LDD motif and the conserved tryptophan are located at the opposite sides of the active sites [12], [13]. It was proposed that the LDD motif guides β-ketoacyl thioester substrates to enter the active site through the entrance close to the NADPH binding site. The orientation of the substrate results in a (*R*)-hydroxyl group. In contrast, the conserved tryptophan in the A-type KR domains leads the substrate to approach NADPH through the other side of the active site and, therefore, exposes the opposite side of the substrate to be attacked by the hydride. The result is a (*S*)-hydroxyl group in the product [11], [12].

Several representative structures of polyketide ketoreductases have been solved in recently years. The KR structures feature a Rossmann fold that is responsible for the binding of the NADPH cofactor [12], [13], [14]. Next to the NADPH binding site, at the bottom of a cleft is the catalytic triad consisting of a tyrosine, a serine and a lysine. As observed in many SDR enzymes, the tyrosine is proposed to be activated by the nearby lysine and donate a proton to the carbonyl oxygen on the keto group [15]. The replacement of the catalytic tyrosine by a phenylalanine abolishes the enzymatic activity [16]. On the top of the active site are the lid helix and the LDD loop that regulate the transportation of substrates and products. In the closed form, the lid helix makes extensive contacts with the residues in the active site, which provides the closing power for the lid structure [13]. Some ketoreductases, such as tylosin ketoreductase (TylKR1) and ketoreductase from the erythromycin synthase (EryKR1), have two subdomains. One subdomain has catalytic activity while the other subdomain has only structural roles [11], [12], [13]. The structural subdomain is involved in the dimerization of KR domains [11]. Through dimerization, two KR domains form a four-subdomain complex. Some KRs only have one domain that contains the active site [14]. However, the single-domain KRs form tetramers that have similar configuration as those four-subdomain KR dimers.

**Figure 1:**
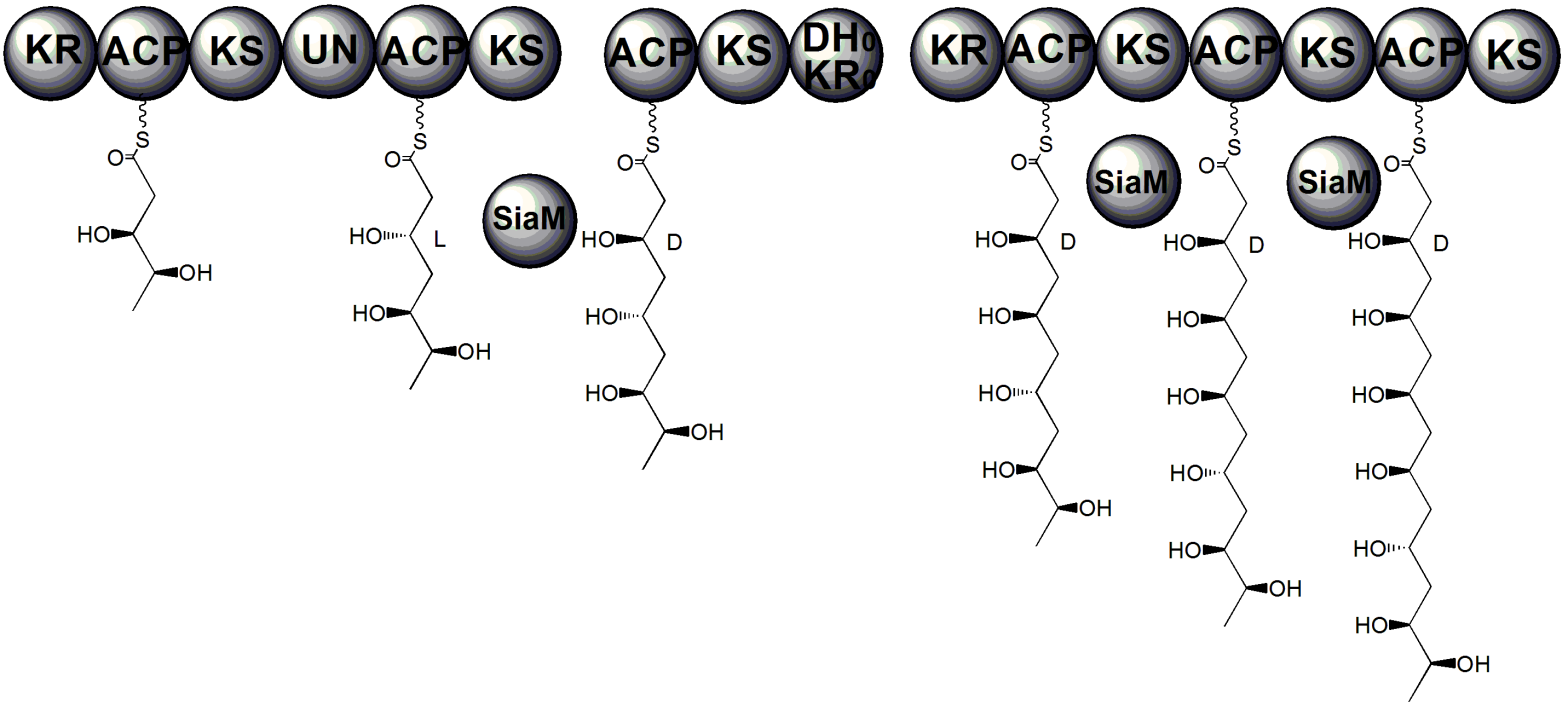
The proposed reactions catalyzed by SiaM. SiaM is proposed to reduce the β-ketoacyl intermediates with various lengths. It is used iteratively along the biosynthetic pathway of polyketide dimer SIA7248 [17].

Most KRs target substrates with a certain length. In the biosynthetic pathways of polyketides, there are usually several KRs each of which is responsible for reducing one keto group on the backbone. However, a recent study has shown that, in the biosynthesis of a symmetric polyketide dimer SIA7248, a *trans-acting* KR SiaM iteratively reduces the β-ketoacyl intermediates attached to different modules of the PKS (Figure 1) [17]. Here, we report the crystal structure of SiaM. Structural analysis indicates that the overall structure resembles those of other KRs. However, significant disparity can be found in the conserved LDD motif. In SiaM, it is replaced with IRD motif. In addition, PISA analysis shows that SiaM forms a tetramer. Several aromatic residues are found in the tetrameric interfaces, which have aromatic stacking interactions with the aromatic residues in the neighboring protomers. Mutagenesis studies performed on the aromatic residues show that these sites are important for maintaining the protein structure.

## Materials and Methods

### Construction of Expression Vectors

The DNA segment of *siam* was amplified from the genome of *Streptomyces* sp. A7248. It was cloned into the pET28a between *Nde*I and *EcoR*I sites. The single-point mutations were introduced by PCR-based site-directed mutagenesis method. The primers used are listed in Table S1. Twelve mutants were constructed including F123A/L/E, Y111A/L/E, F227A/L/E and Y235A/L/E (Table S1). The resulting plasmids were amplified in DH5α. The mutations were then confirmed by DNA sequencing.

### Protein Expression and Purification

Wild-type SiaM and mutants were expressed in *Escherichia coli* BL21 (DE3) pLyS. The protein expression was induced with 0.3 mM Isopropyl β-D-1-thiogalactopyranoside (IPTG), and the incubation lasted for 18 hours at 16°C. The cells were collected by centrifugation at 5,000 rpm, 4°C for 10 min, and then suspended in 20 mL buffer containing 20 mM Tris, 0.5 M KCl, pH 8.3. The cells were lysed by sonication, and the cellular debris was removed by centrifugation at 12,000 g for 1 hour. The recombinant SiaM was purified by chromatography with Ni-NTA resin. The protein was washed with wash buffer (30 mM imidazole, 20 mM Tris, 500 mM KCl, pH 8.3) and eluted with elution buffer (200 mM Imidazole, 20 mM Tris, 500 mM KCl, pH 8.3). It was then further purified with chromatography on a Superdex 75 column (16/60, G. E. Healthcare) equilibrated with 500 mM KCl, 20 mM Tris, pH 8.3.

### Crystallization and Data Collection

Crystallization was carried out using sitting drop vapor diffusion method by mixing 1 µL protein solution with 1 µL precipitant solution. The concentration of SiaM for crystallization was 16 mg/mL. The crystals were observed in the condition containing 1.5 M ammonium sulfate, 0.1 M Tris pH 8.5 and 12% v/v glycerol after one week at 4°C, and reached their maximum size in ten days. The crystals were soaked in the cryoprotectant containing the mother liquor plus 15% ethylene glycol (Acros) before frozen in liquid nitrogen.

Diffraction data were collected at 100 K with an ADSC Q315 CCD detector on beam-line BL17U, SSRF, Shanghai, China. A total of 180 images were collected with one degree oscillation per image with exposure time of 1 second. The data set was indexed with XDS package and scaled with Scala, the mean mosaicity of the dataset is 0.91 [18], [19]. The structure was solved by molecular replacement with the program Phaser using the 3-oxoacyl-(acylcarrier protein) reductase (BA3989) from *Bacillus anthracis* (PDB ID: 2UVD) as the search model [20]. Auto-model building was performed using Phenix which was followed by the manual model building performed by Coot [21]. Along with the manual building process, the refinement was done with Phenix [22]. The model quality was evaluated with MolProbity [23]. Data parameters and refinement statistics are summarized in Table 1. The graphics were generated with PyMol [24]. The interface and assembly information were obtained with service PISA at European Bioinformatics Institute [25]. The atomic coordinates and the structure factors of SiaM have been deposited in the Protein Data Bank with the accession code 3WOH.

**Table 1:** Data collection and refinement statistics.

Data collection	
Resolution range (Å)^1^	51.27–2.5 (2.589–2.5)
Space group	P 4_2_ 2_1_ 2
Cell dimensions	
a, b, c (Å)	56.42 56.42 122.8
α, β, γ (°)	90 90 90
Total reflections	40463 (4024)
Unique reflections	7354 (716)
Multiplicity	5.5 (5.6)
Completeness (%)	99.49 (99.86)
Mean I/sigma (I)	15.76 (2.12)
Wilson B-factor (Å^2^)	46.93
R_merge_	0.09461 (0.8619)
Refinement	
R_work_ 2	0.2367 (0.3505)
R_free_ 2	0.2736 (0.3706)
Number of non-hydrogen atoms	1722
Macromolecules	1707
Water	15
Protein residues	235
R.m.s deviations	
Bond lengths (Å)	0.006
Bond angles (°)	1.05
Ramachandran plot^3^	
Favored (%)	98
Outliers (%)	0
Average B-factor (Å^2^)	67.50
macromolecule	67.60
solvent	45.50
PDB ID	3WOH

1 Data in the parenthesis was calculated according to the highest resolution shell.

2 R-factor = (Σhkl||Fo|-|Fc||)/Σhkl|Fo| where Fo and Fc are the observed and calculated structure factors respectively. R_free_ was calculated with a randomly selected 5% subset that was excluded from the refinement process.

3 MolProbity was used to calculate the statistics of Ramachandran plot.

### Enzymatic Characterization of SiaM and Mutants

Assays for the functions of SiaM and mutants were carried out at 30°C overnight with a reaction volume of 100 µl. It was performed in the presence of 20 mM Tris buffer (pH 8.3), 3 mM NADPH, 4 mM SiaM, and 1 mM 3-keto- butanoyl-SNAC (dissolved in DMSO). The solution was extracted with 0.2 mL ethyl acetate after reaction. Then the organic phase was separated, dried, re-dissolved in 20 µl of methanol, and analyzed with HPLC. HPLC was carried out on an America SSI HPLC Series 2300 using an Apollo C18 column (250×4.6 mm, 5 µm). The mobile phase used for HPLC was composed of solvent A (H_2_O containing 0.1% formic acid) and solvent B (CH_3_CN). The analytical HPLC was eluted with a linear gradient of 5% to 40% solvent B from 0 to 15 min, 40% to 100% solvent B from 15 to 22 min, 100% solvent B kept for 5 min, 100% to 5% solvent B from 27 to 28 min, and followed by 5% solvent B for 6 min. The flow rate was 0.6 mL/min and UV detection was set at 240 nm.

### Small-angle X-ray Scattering

SiaM was purified by chromatography with Ni-NTA resin, and then further purified with gel-filtration on a Superdex 75 column (16/60, G. E. Healthcare) equilibrated with 10 mM Na_2_HPO_4_, 1.8 mM KH_2_PO_4_, 400 mM Na_2_SO_4_, pH 8.3. After purification, SiaM was concentrated by ultrafiltration (MilliPore), and the protein concentration was determined by the absorption at 280 nm using an Eppendorf spectrophotometer.

Small-angle X-ray scattering (SAXS) data from SiaM were collected on a Anton Paar SAXSess mc^2^ system (Wuhan Institute of Physics and Mathematics, Chinese Academy of Sciences). The buffer scattering was determined by subtracting the scattering of the empty cuvette from that filled with PBS buffer (10 mM Na_2_HPO_4_, 1.8 mM KH_2_PO_4_, 400 mM Na_2_SO_4_, pH 8.3) at 4°C. The measurements of the protein and buffer scattering were repeated in three separate experimental sessions and the results were averaged. The data were processed by ATSAS software package and the data quality is assessed with program AUTORG [26]. The structures of SiaM monomer, dimer and tetramer were fitted to the saxs data with the program CRYSOL [27]. And the oligomeric state of the protein was analyzed with program OLIGOMER [28].

### Spectroscopic Measurements

Fluorescence measurements were taken with a Jasco FP-6500. The proteins were kept in 1.8 mM KH_2_PO_4_ (pH 8.3), 10 mM Na_2_HPO_4_, 150 mM Na_2_SO_4_ with a concentration of 0.6 mg/mL. And the spectra were recorded with a scanning speed of 500 nm/min at room temperature. All measurements were assayed for three times and subtracted by the solvent spectra. Then the emission spectra were recorded from 280 nm to 450 nm at an excitation wavelength of 270 nm.

## Results

### The Overall Structure of SiaM

**Figure 2:**
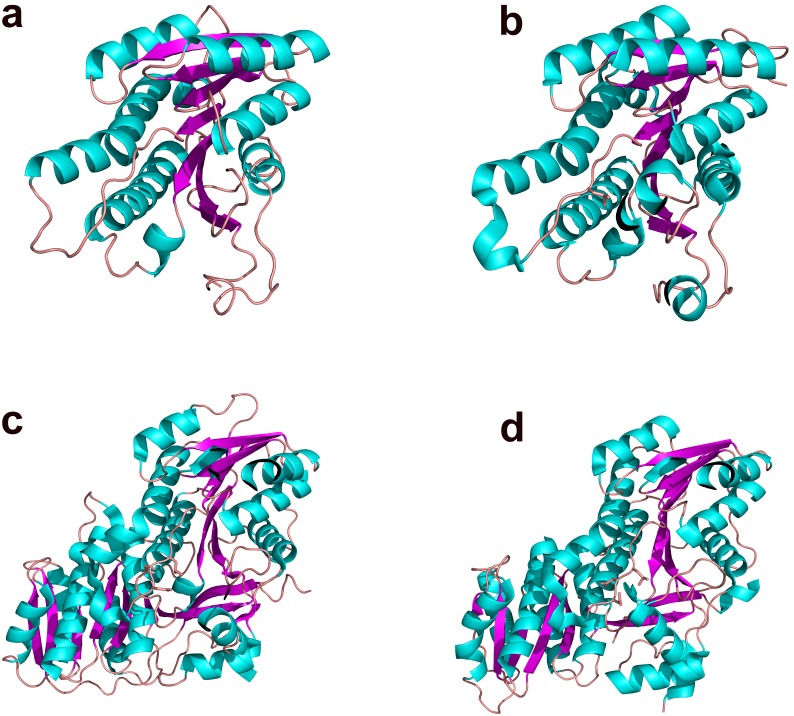
The overall structures of several polyketide KRs. The ribbon diagrams are colored by secondary structures. The helices are in cyan; the sheets are in magenta and the loops are in orange. (a) SiaM from *Streptomyces* spp. A7248; (b) ActKR from *Streptomyces coelicolor*; (c) TylKR1 from *Streptomyces fradiae*; (d) EryKR1 from *Streptomyces erythraea*.

**Figure 3:**
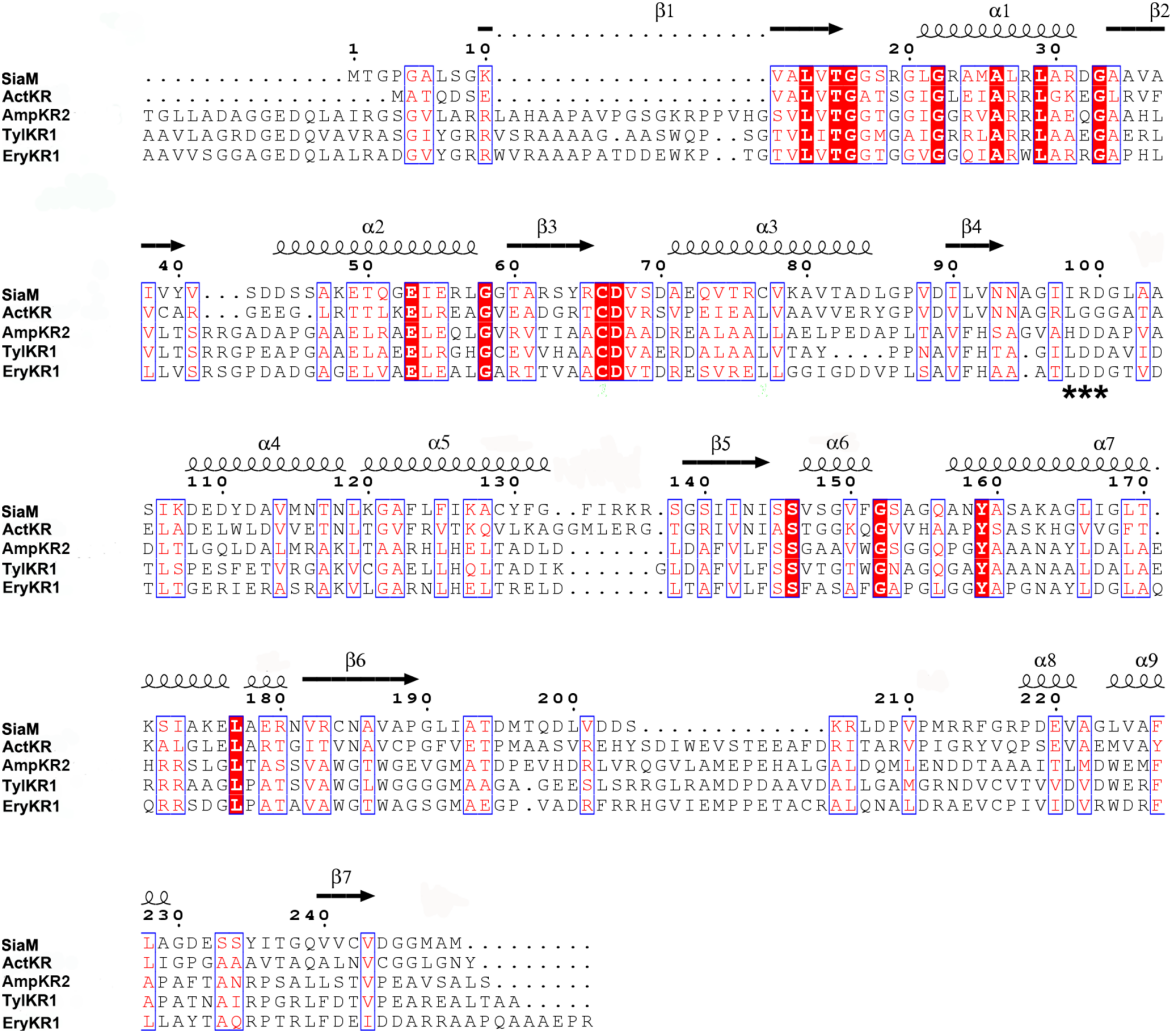
Structure-based sequence alignment of KRs from different sources. The sequence of SiaM is aligned with sequences of ActKR from *Streptomyces coelicolor*, TylKR1 from *Streptomyces fradiae*, EryKR1 from *Streptomyces erythraea* and AmpKR2 from *Streptomyces nodosus*. The sequences are annotated with corresponding secondary structures in SiaM. Arrows represent β-strands and helices represent α-helices. The conserved residues are colored in red. The LDD motif is marked by asterisks.

**Figure 4:**
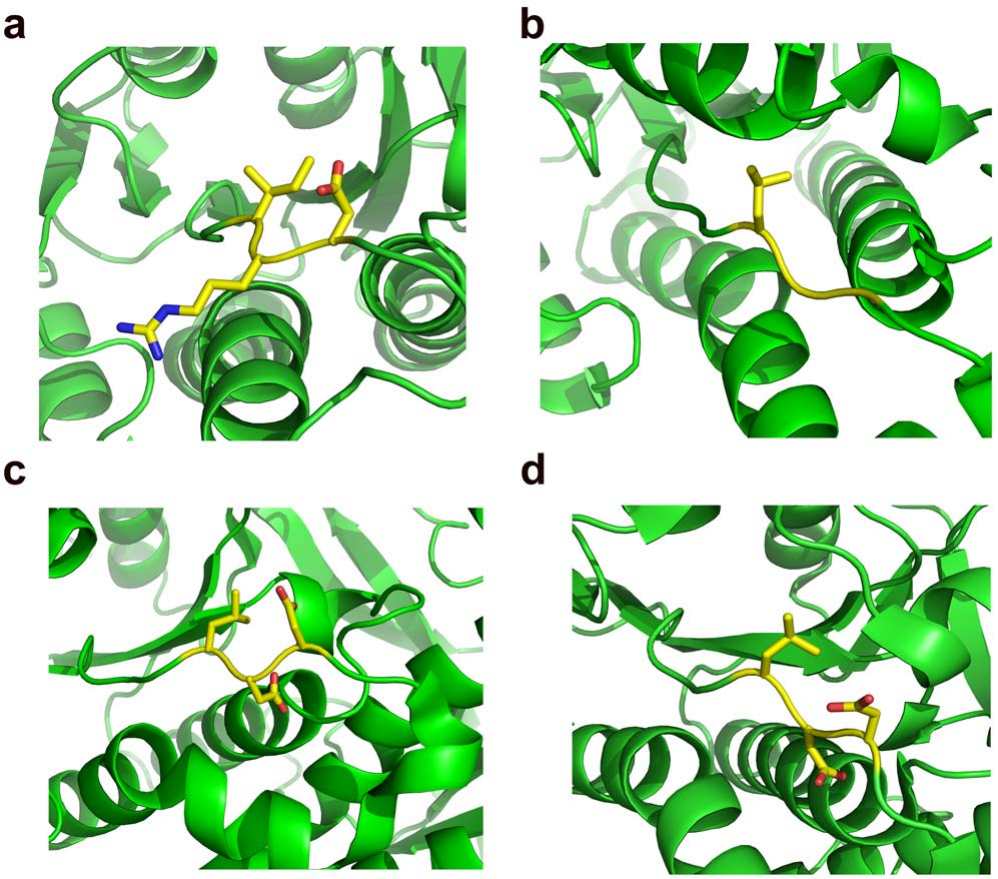
The comparison of LDD and LDD-like motifs in KRs. The LDD and LDD-like motifs are shown as sticks and colored by elements. The rest of the molecules are shown as ribbons and colored in green. (a) The IRD motif in SiaM. The IRD motif is a degenerate form of LDD motif, with a hydrophobic residue occupying the first position and an aspartic acid occupying the third position. (b) The LGG motif in ActKR. (c) The LDD motif in TylKR1. (d) The LDD motif in EryKR1.

The overall structure of SiaM resembles the consensus structure of KRs involved in the fatty acid biosynthesis as well as polyketide biosynthesis [10], [29]. It harbors a Rossmann fold which is responsible for the binding of NADPH molecules [30]. The Rossmann fold has a 7-strand β sheet at center and 9 α helices surrounding it (Figure 2). On the side of Rossmann fold lies the active site cleft where the ACP engages the KR and positions the substrate adjacent to the nicotinamide moiety. The structure of SiaM is highly similar to that of actinorhodin KR (ActKR), a KR from type II PKS (type II KR) [10]. The superposition of SiaM with the ActKR results in a root-mean-square deviation of 1.12 Å over 240 C_α_ carbons. Although there is one SiaM molecule in each asymmetric unit, it forms a tetramer through symmetry operations. Similar composition is found in many KRs, such as various FabG and ActKR [14], [29], [31], [32]. In type I KRs (KRs from type I PKSs), the LDD motif is proven to be the determinant of stereospecificty as well as stereochemistry [10], [13]. Sequence alignment of this subgroup shows that L in the LDD motif can be replaced with other hydrophobic residues, the first D can be replaced with other acidic residues and the second D is strictly conserved [9]. Superposition of SiaM, EryKR1, TylKR1 structures indicates that a variant form of LDD motif (IRD) can be found at the same location in SiaM (Figure 3). Consequently, the majority of SiaM product has (*R*)-configuration on the β carbon [17]. Noticeably, the second position of the LDD motif is occupied by a basic arginine residue, which has not been reported before. The orientation of the first residue in the LDD motif is conserved in various type B KRs. The hydrophobic side chains point towards the nicotinamide moiety and guide the polyketide chain to the active site [13]. The second D in the LDD motif is responsible for closing the active site through hydrogen bonding with residues on the lid helix. In contrast, the function of the first D in LDD motif has not been assigned yet. Also this position is less conserved than other two positions [9]. Consistently, the orientation of Arg99 in SiaM doesn’t match those of corresponding residues in TylKR1 and EryKR1 (Figure 4) [12], [13]. Instead of pointing towards the active site, the Arg99 is positioned towards solvent. However, the altered orientation of this residue doesn’t seem to affect the function of the enzyme.

### The Active Site Configuration

**Figure 5:**
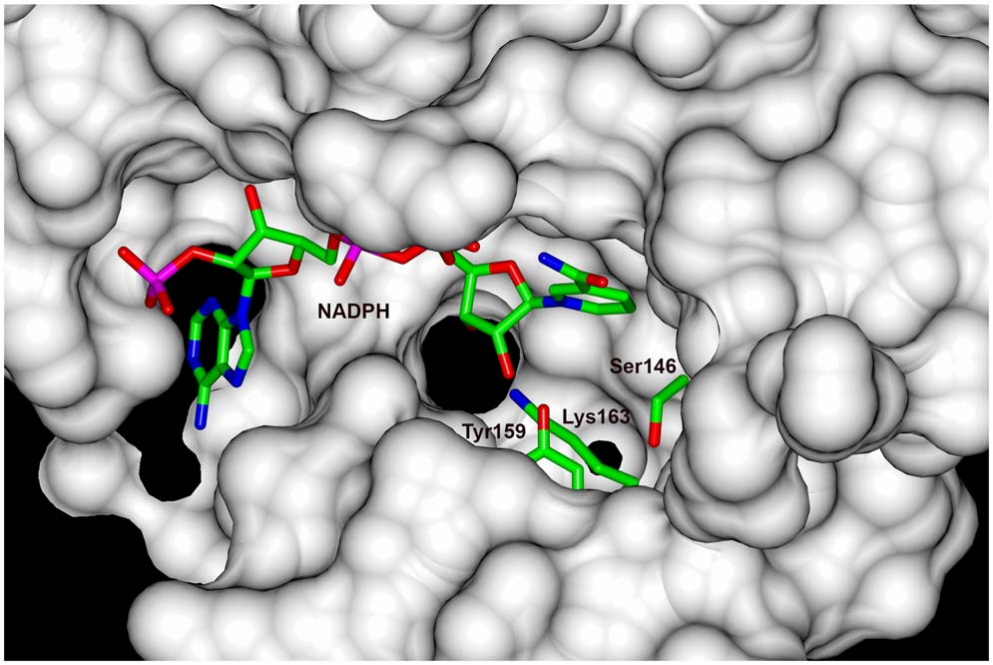
The catalytic triad and the NADPH binding site. The NADPH binding site is predicted by overlapping the structure of SiaM and the structure of ActKR/NADPH complex. The NADPH molecule and the catalytic triad (Ser146, Tyr159, Lys163) are shown in sticks. The rest of SiaM is displayed as surface and colored in light gray.

The binding site of NADPH is highly conserved among different KRs. Although SiaM crystallized without NADPH, the superposition of SiaM and other KR/NADPH complex structures indicates that NADPH binding cavity is present at the same location in the SiaM structure (Figure 5). Therefore, SiaM and NADPH should have similar binding pattern as other KRs. Close to the NADPH binding site are the catalytic residues (Ser146, Tyr159, Lys163) that are conserved among KRs from various sources (Figure 5). Also found in the active site is the Asn118, which is found in type I KR Tylk1, EryKR1 and type II KR ActKR. As suggested in the previous study, the reduction is initiated by the nucleophilic attack of carbonyl carbon with the hydride from NADPH. The resulting alkoxide deprotonates adjacent tyrosine. Then the proton is reinstalled by the lysine [14]. Although the substrate of SiaM is linear, which implies it belongs to type I group of KRs, the arrangement of Lys163 and Asn118 is opposite of that in the TylKR1 and EryKR1. In addition, a water molecule is found in the vicinity of conserved Lys163, which was proposed to be involved in the proton relay [31].

### The Quaternary Structure of SiaM

**Figure 6:**
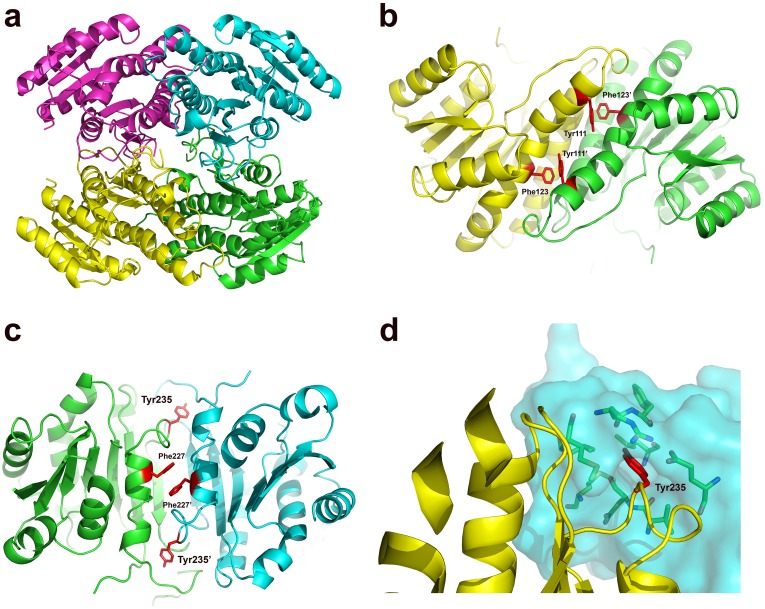
The composition of the SiaM tetramer. The SiaM tetramer is constructed through symmetry operations. (a) The square-shaped SiaM tetramer. Each protomer is colored in red, yellow, green and cyan separately. (b) The aromatic stacking interactions in the N-terminal interface. The Phe123, Tyr111 in one protomer and Phe123′, Tyr111′ in the other protomer are shown as sticks and colored in red. The rest of the protomers are colored in yellow and green separately. The aromatic residues form T-shaped aromatic stacking interactions. (c) The aromatic stacking interaction in the C-terminal interface. The Phe227, Tyr235 in one protomer and Phe227′, Tyr235′ in the other protomer are shown as sticks and colored in red. The rest of the protomers are colored in green and cyan separately. The Phe227 and Phe227′ form a parallel-displaced aromatic stacking interaction. (d) The interface formation around Tyr235. The Tyr235 residue is colored in red and shown as sticks. The rest of the molecule is shown in ribbon. The neighboring protomer is shown as surface. A deep cavity is clearly visible at the Tyr235 binding site. The residues in the cavity are shown as sticks.

SiaM forms a tetramer through symmetry operations (Figure 6a). Two kinds of interfaces are found in the tetramer. One consists of α4 and α5 helices of neighboring protomers. The other is formed by a α9 helix from each protomer. The interactions between α4 and α5 helices include hydrogen bonding (Lys120/Asp112’; Ile106/Lys127’) and hydrophobic interactions involving numerous hydrophobic residues. In this interface, the α4 and α5 helices from different protomers are positioned in anti-parallel directions. As a result, the Tyr111 is located in the vicinity of the Phe123, which form aromatic stacking between them (Figure 6b and Figure S1). Similarly, in the other interface of the tetramer, the aromatic stacking is found between Phe227 residues from neighboring protomers (Figure 6c and Figure S1). In addition, Tyr235 inserts into the binding pocket in the opposite protomer and forms hydrogen bonds with the backbone carbonyl groups (Figure 6d and Figure S1). In the sequence alignment of ActKR and SiaM, the position 111 and 123 (as numbered on SiaM) are occupied with aromatic residues, which is consistent with the similar interfaces on these two proteins [14]. The position 227 is occupied by an aromatic residue in all five polyketide KRs with structures available (Figure 3). The structural analysis of these KRs shows that the aromatic stacking is a common feature in their interfaces [11], [12], [13], [14]. In contrast, Tyr235 is not conserved. The interaction Tyr235 and neighboring protomer is unique for SiaM.

**Figure 7:**
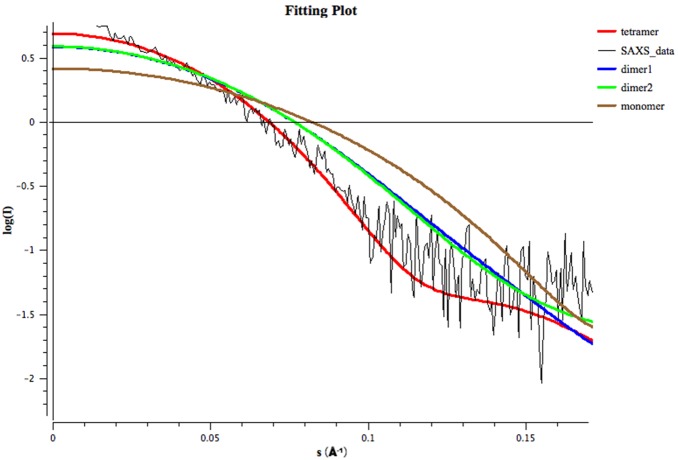
Small-angle X-ray scattering. The theoretical scattering data were calculated based on the coordinates of the SiaM monomer, dimer1, dimer2 and tetramer. The theoretical data were compared with experimental SAXS data using program CRYSOL. The fitting curves of monomer (brown), dimer1 (green), dimer2 (blue) and tetramer (red) are superposed with the SiaM scattering data (black).

To investigate the oligomeric state of SiaM in solution, a small-angle X-ray scattering experiment was performed. The data were processed with ATSAS package. As shown in the output of the program GNOM, the Rg of the possible SiaM complex is 3.45 nm, the I_0_ is 5.06, the Dmax is 10.76 nm and the Porod volume is 201.96 nm^3^ [33]. The tetramer and two possible dimers (dimer1, dimer2) were generated based on the SiaM crystal structure through symmetry operations. The theoretic scattering curves of SiaM monomer, dimer1, dimer2 and tetramer were calculated with the program CRYSOL. Then the curves were compared with the experimental SAXS data (Figure 7). The results indicate that the Chi values for the fitting of tetramer, dimer1, dimer2 and monomer are 0.785, 1.514, 1.485 and 2.856 respectively. Therefore, the tetrameric formation is the best fit for the scattering data. Also the data were analyzed with program OLIGOMER to find the possible mixture of SiaM monomer, dimer and tetramer. The results indicate clearly that 100% of the protein is in a tetrameric state.

### The Putative ACP Binding Site

The docking of ACP is required to deliver the nascent polyketide chains to various KRs [34], [35], [36]. The positioning of polyketide chain in the active site, which is linked to ACP through a phosphopantetheinyl group, is thought to determine the stereochemistry of ketoreduction reaction [11], [12]. The binding interface in ACP has been predicted with molecular docking [11], [14]. It contains a conserved hydrophobic residue followed by a phosphopantetheinylated serine. This forms the hydrophobic interactions between the ACP and the KR [11]. In addition, the majority of the binding force is provided by the hydrophilic interactions due to the large number of charged residues on the ACP. In case of ActKR, several arginine residues (R177, R216, R220) form hydrogen bonds with aspartic acid residues on the surface of ACP [14]. Also arginine residues (R149 and R172) in *E. coli* FabG are proved to be important in the binding of ACP [37]. However, none of these arginine residues is conserved in SiaM, which implies different ACP binding pattern. An invariant element of ACP binding site is the lid helix. In the docking result of ACP to ActKR and AmpKR, the lid helix (or equivalent region) is the contacting site for ACP. The lid helices are the most flexible parts in the available KR structures, which have poorly-defined regions or elevated B factors [11], [14]. In SiaM structure, this region is only partially visible. Unlike many other KRs, which contact only one ACP, the iterative nature of SiaM implies that it works with multiple ACPs each of which carries a polyketide chain with a certain length. The binding of SiaM and ACPs may have an induced-fit mechanism. Consequently, the lid helix region stays highly flexible, which makes it easy to accommodate different ACP partners.

### Intrinsic Fluorescence Assay

**Figure 8:**
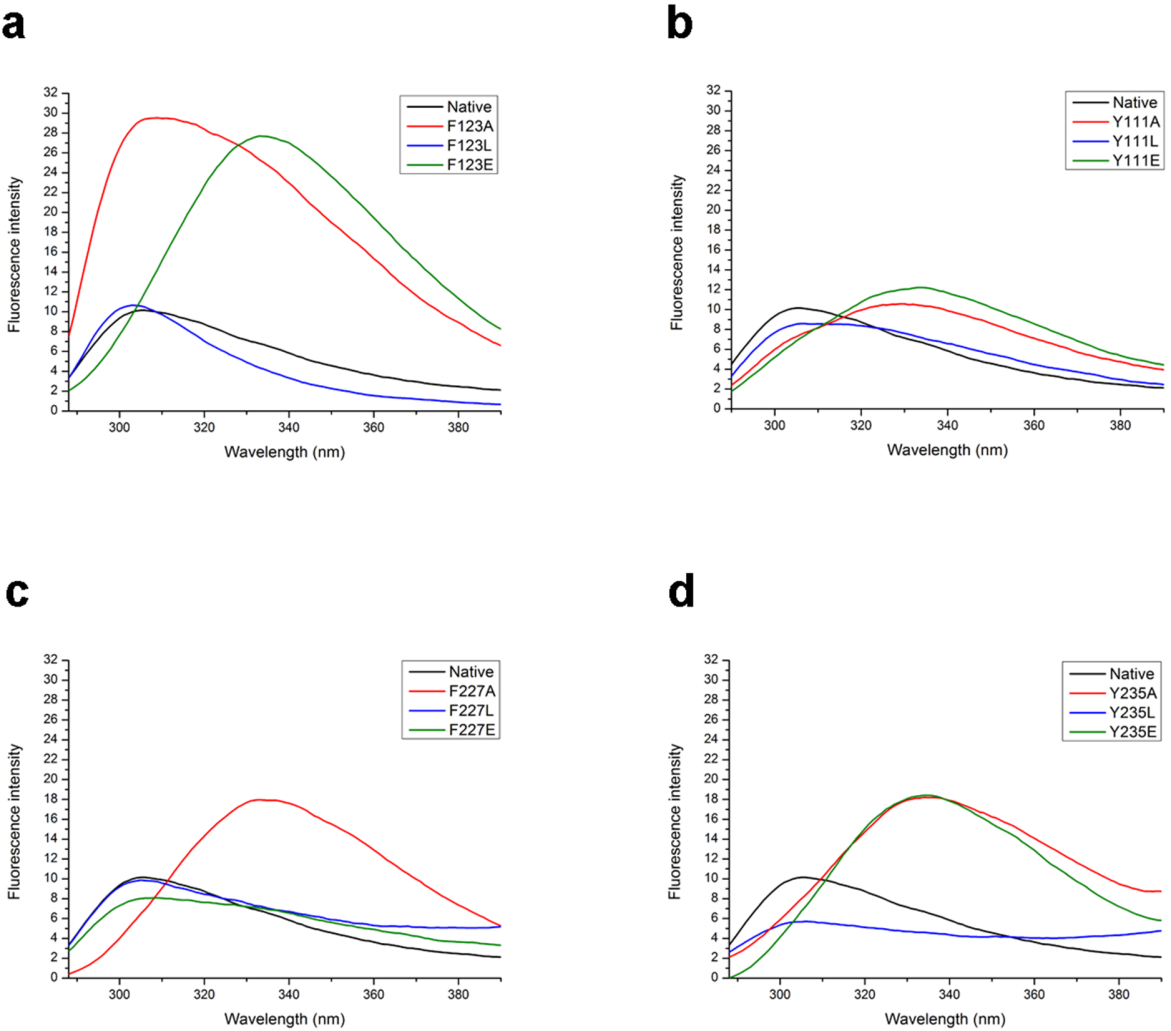
Fluorescence scans of wild-type SiaM and the mutants. Fluorescence scans were used to monitor the tertiary structures of SiaM and its mutants. The fluorescence intensity is shown in arbitrary units. (a) Spectra of the mutants at F123. (b) Spectra of the mutants at Y111. (c) Spectra of the mutants at F227. (d) Spectra of the mutants at Y235.

Intrinsic fluorescence of aromatic residues was used as a probe to study the tertiary structures of wild-type SiaM and mutants. Usually, the main portion of fluorescence comes from the tryptophan residues. However, SiaM doesn’t possess any tryptophan residue in its sequence. The recorded fluorescence is the emission from tyrosine and phenylalanine residues. Although tyrosine and phenylalanine produce much weaker fluorescence than tryptophan, SiaM has 7 phenylalanine and 6 tyrosine residues. The cumulative effect leads to the fluorescence at recordable level. The wild-type protein has a maximum emission at 305 nm. The substitutions with leucine at 3 positions (F123, Y111, F227) yield similar spectra as that of the wild-type protein, indicating no significant change in their tertiary structures (Figure 8). They also indicate that the removal of a single aromatic residue does not affect the spectra. In contrast, the substitution with alanine results in noticeable change in the spectra as far as fluorescence intensity and peak position. This is consistent with the fact that alanine substitution, with most of the side-chain absent, causes large disturbance to the protein structure. The substitution with glutamic acid has mixed results. It leads to altered spectra for mutants F123E, Y111E, Y235E, while the spectrum of F227E is almost the same as that of wild-type protein. Interestingly, the fluorescence intensities of several mutants (F123A, F123E, F227A, Y235A, Y235E) increase significantly,indicating these mutants are in partially folded states.

### Enzymatic Activities of the SiaM Mutants

**Figure 9:**
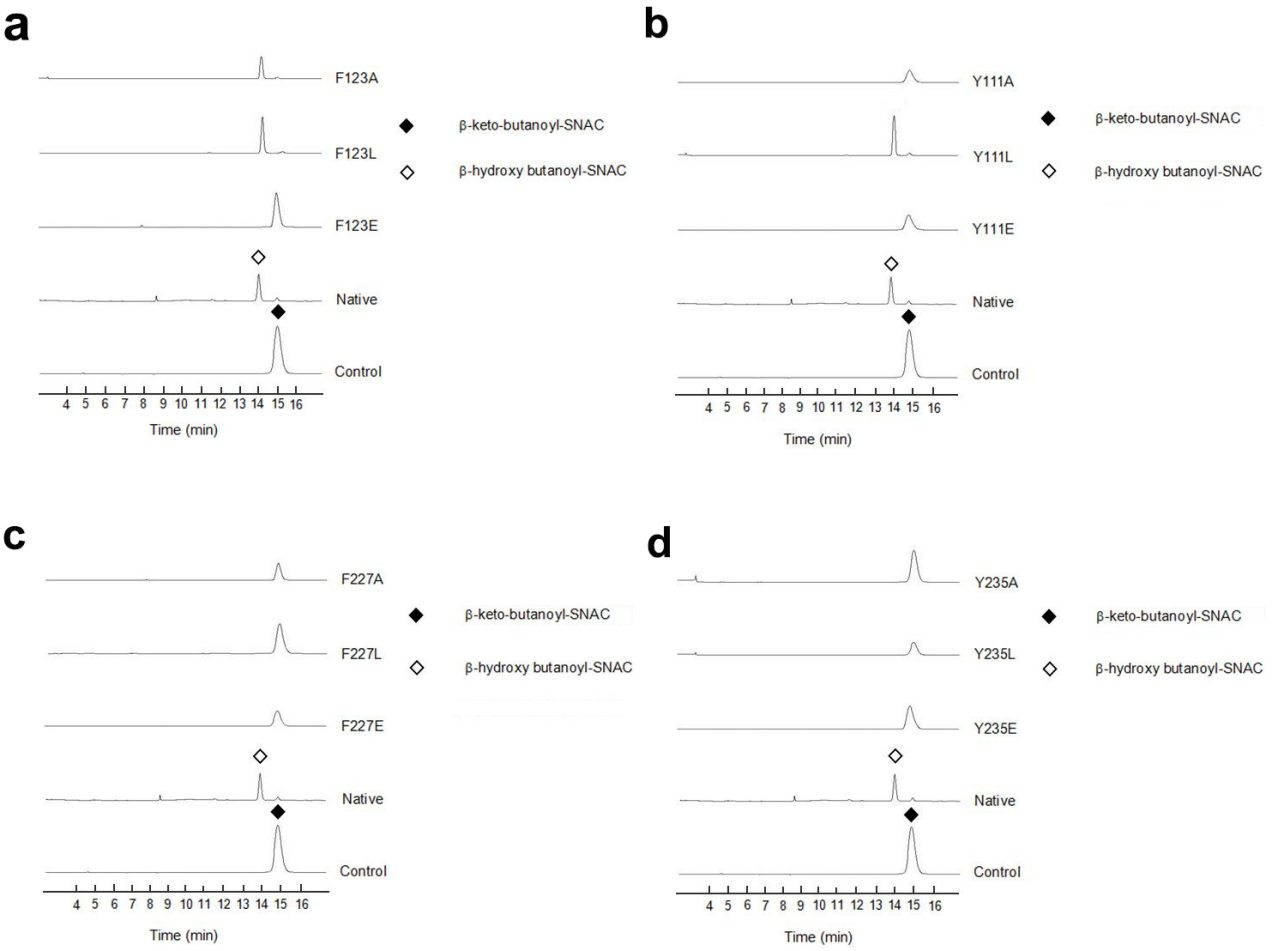
Enzymatic activity assays of wild-type SiaM and its mutants. The assays were performed with the substrate mimic β-keto-butanoyl-SNAC. The product and substrate were separated with HPLC and monitored at UV 240 nm. (a) Assays with mutants at F123. (b) Assays with mutants at Y111. (c) Assays with mutants at F227. (d) Assays with mutants at Y235.

The enzymatic activities of SiaM mutants were studied as reported before (Figure 9) [17]. In one interface, the mutations (Y111A, Y111E, F123E) abolish the enzymatic activities, while mutations (Y111L, F123A, F123L) do not affect the activity. Because the substitutions with leucine keep the hydrophobic effects but remove the aromatic stacking, the hydrophobic effects at these positions are important to the enzymatic activity while the aromatic stacking is not essential. In contrast, in the other interface of the tetramer, all mutations (F227A, F227L, F227E, Y235A, Y235L, Y235E) abolish the enzymatic activity. Therefore, in this interface, the aromatic stacking is essential for the enzymatic activity.

## Discussion

Ketoreductases are a group of enzymes ubiquitously distributed in the polyketide and fatty acid biosynthesis pathways. Due to their importance, a lot of research has been conducted to study their structures, functions as well as catalytic mechanisms [11], [13], [38]. In this SiaM structure, a water molecule is found next to the catalytic lysine (K163). In the other crystal form of SiaM, three more water molecules are found in the active site that form a water chain (Data not shown). Similar water chains have been located in other KRs such as FabG and ActKR [14], [32]. After searching the PDB database, it was found that the water molecules can be found at the same locations in the structures of plant fatty acid KR from *Brassica napus* (PDB ID 1EDO) and glucose dehydrogenase from *Bacillus megaterium* (PDB ID 1GCO) [39], [40]. It is possible that this water chain is common among SDRs. It was proposed that the deprotonated lysine 163 is replenished through a proton relay network including ribose hydroxyl groups on NADPH and the water chain [14], [31], [41]. Interestingly, the water chain is buried deep inside of the protein. It is extended from the catalytic lysine to the hydrophobic core of the enzyme and doesn’t communicate with the solvent. If protons are delivered through this chain, additional residues are needed to carry the protons to the solvent.

Aromatic stacking has proven to be an important factor for stabilizing nucleic acids structures due to the extensive stacking effects between various bases [42]. Frequently, aromatic stacking interactions are found in the interfaces of protein/nucleic acid complexes. The aromatic residues at the binding site form pi-pi stacking with nucleic acids and achieve specific recognition between the binding partners [43], [44]. The aromatic stacking interactions between proteins are relatively rare because the large hydrophobic aromatic residues are seldom located on the surface of proteins. However, the aromatic clasps have been found in the interfaces of several enzymes, such as GSTs, TIMs and FabGs [32], [45], [46], [47]. Two types of aromatic stacking exist in protein structures. One is parallel-displaced and the other is T-shaped [48]. Usually, one kind of aromatic stacking is found in the interfaces in the protein oligomers [45], [49]. Interestingly, both types of stacking configurations are present in the interfaces of SiaM. Consequently, the solvation free energy gain upon formation of the SiaM tetramer is −69.8 kcal/mol and the buried surface is 12270 Å^2^. In contrast, the ActKR tetramer, which is the homolog of SiaM, has buried surface of 21980 Å^2^, while its solvation free energy gain (−57.3 kcal/mol) is significant less than that of SiaM [25]. Also found in the interface is a N-H/pi interaction between arginine and tyrosine residues(Figure 6d). The N-H/pi interaction is classified as a hydrogen bond. Its bonding energy (−3.5 kcal/mol) is approximately half that of regular O-H/O type hydrogen bonds (−7.8 kcal/mol) [50], [51], [52]. Therefore, it can be a good contributing factor for the interface stability. The N-H/pi interaction has proven to be important in the folding of bovine pancreatic trypsin inhibitor. In the late stage of folding, the interaction between Tyr35 and Gly37 prevents the loop from forming nonfunctional conformation [53]. Also, N-H/pi interactions are involved in the ligand recognition of many drug-target proteins [51], [54]. However, N-H/pi interaction at the protein-protein interface is not common. SiaM is a rare example that such interaction is involved in the protomer interaction. The other interaction through aromatic residues is the C-H/pi interaction. Although its bonding energy (−0.88 kcal/mol) is about one tenth of a regular hydrogen bond, it can be a good stabilizing force for the protein structures which sometimes only require a few kcal/mol energy [50].

SiaM sequence was used to search the PDB database. After examining the homologs’ structures, we found that aromatic stacking is a common feature of this group of proteins. However, only parallel-displaced aromatic stacking is found in the interfaces of these proteins except for SiaM. SiaM tetramer possesses a rare T-shaped stacking configuration. Some study has been performed to find the distribution of parallel-displaced and T-shaped stacking in the PDB database. It is found that the majority of them are parallel-displaced, which is consistent with our finding among SiaM and its homologs [48]. A secondary structure comparison was performed on Dali server with the SiaM structure as the template. The results indicate that most proteins with structures homologous to SiaM are 3-oxoacyl-[acyl-carrier-protein] reductases. Structural analysis shows that all the hits with high Z-scores have a tetramer structure that has aromatic stacking in the interfaces. The tetramer seems to be a common composition for 3-oxoacyl-[acyl-carrier-protein] reductases.

## Supporting Information

Figure S1 **The electron-density maps around aromatic residues in the tetrameric interfaces.** The 2Fo-Fc maps are contoured at 1.5 sigma level and clipped around aromatic residues.
(TIF)

Table S1 **Primers for** ***siam*** **gene amplification and site-directed mutagenesis.**
(DOCX)
