# Supplementary figures and images for: Structural Insight into the Tetramerization of an Iterative Ketoreductase SiaM through Aromatic Residues in the Interfaces
Source: PLoS One. 2014 Jun 5;9(6):e97996. doi: 10.1371/journal.pone.0097996 (PMC4046962; doi:10.1371/journal.pone.0097996)

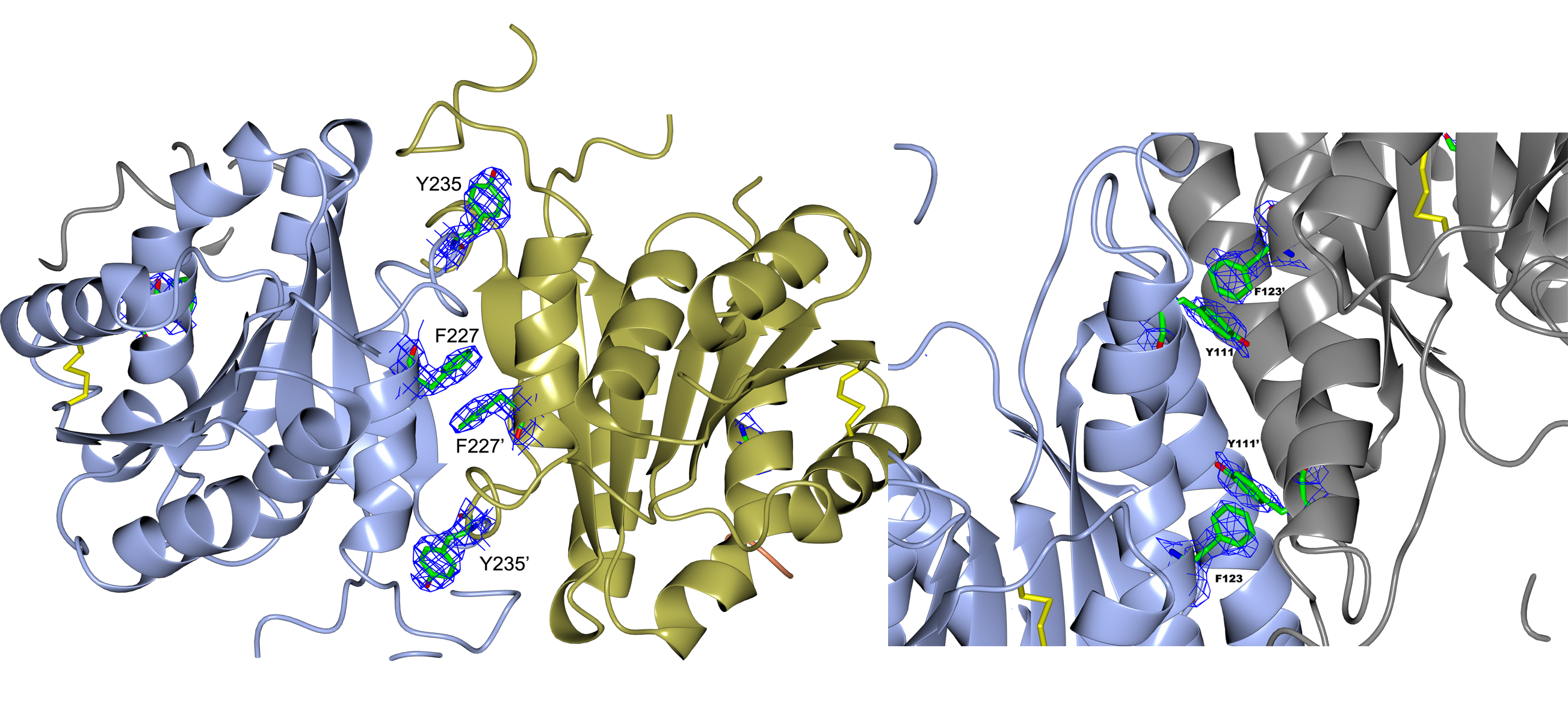

Supplement: Figure S1 — The electron-density maps around aromatic residues in the tetrameric interfaces. The 2Fo-Fc maps are contoured at 1.5 sigma level and clipped around aromatic residues. (TIF) [file pone.0097996.s001.tif]
