# Supplementary material for: Structural Insight into the Tetramerization of an Iterative Ketoreductase SiaM through Aromatic Residues in the Interfaces
Source: PLoS One. 2014 Jun 5;9(6):e97996. doi: 10.1371/journal.pone.0097996 (PMC4046962; doi:10.1371/journal.pone.0097996)
Supplement: Table S1 — Primers for siam gene amplification and site-directed mutagenesis. (DOCX) [file pone.0097996.s002.docx]

**Table S1** Primers for SiaM gene amplification and site-directed mutagenesis.

| Name of sequence | Sequence (5’-3’) | Name of sequence | Sequence (5’-3’) |
| --- | --- | --- | --- |
| F123A | CTCAAGGGCGCGGCCCTGTTCATCAAGGCCT | Y111A | CATCAAGGACGAGGACGCCGACGCGGTGATGAAC |
| F123A anti | AGGCCTTGATGAACAGGGCCGCGCCCTTGAG | Y111A anti | GTTCATCACCGCGTCGGCGTCCTCGTCCTTGATG |
| F123L | CTCAAGGGCGCGCTCCTGTTCATCAAGGCCT | Y111L | CATCAAGGACGAGGACCTCGACGCGGTGATGAAC |
| F123L anti | AGGCCTTGATGAACAGGAGCGCGCCCTTGAG | Y111L anti | GTTCATCACCGCGTCGAGGTCCTCGTCCTTGATG |
| F123E | CTCAAGGGCGCGGAACTGTTCATCAAGGCCT | Y111E | CATCAAGGACGAGGACGAAGACGCGGTGATGAAC |
| F123E anti | AGGCCTTGATGAACAGTTCCGCGCCCTTGAG | Y111E anti | GTTCATCACCGCGTCTTCGTCCTCGTCCTTGATG |
| F227A | GGTCTGGTCGCCGCCCTGGCCGGCGACGAG | Y235A | GACGAGAGCTCGGCCATCACCGGGCAGGT |
| F227A anti | CTCGTCGCCGGCCAGGGCGGCGACCAGACC | Y235A anti | ACCTGCCCGGTGATGGCCGAGCTCTCGTC |
| F227L | GGTCTGGTCGCCCTCCTGGCCGGCGACGAG | Y235L | GACGAGAGCTCGCTCATCACCGGGCAGG |
| F227L anti | CTCGTCGCCGGCCAGGAGGGCGACCAGACC | Y235L anti | ACCTGCCCGGTGATGAGCGAGCTCTCGTC |
| F227E | GGTCTGGTCGCCGAACTGGCCGGCGACGAG | Y235E | GACGAGAGCTCGGAAATCACCGGGCAGGT |
| F227E anti | CTCGTCGCCGGCCAGTTCGGCGACCAGACC | Y235E anti | ACCTGCCCGGTGATTTCCGAGCTCTCGTC |
| SiaM | CGCCATATG ATGACGGGGCCCGGAGCCCTCA | SiaM anti | CGGAATTC TTATCACATCGCCATGCCGCCGTCCACA |
